# Patient cost-sharing for ambulatory neuropsychiatric services in Abu Dhabi, UAE

**DOI:** 10.1186/s13033-016-0066-6

**Published:** 2016-04-21

**Authors:** Samer Hamidi, Yousef Abouallaban, Sultan Alhamad, Aizhan Meirambayeva

**Affiliations:** School of Health and Environmental Studies, Hamdan Bin Mohammed Smart University, Dubai, United Arab Emirates; American Center for Psychiatry and Neurology, Abu Dhabi, United Arab Emirates; Windsor, Canada

**Keywords:** Abu Dhabi, United Arab Emirates, Neuropsychiatric disorders, Cost-sharing, Insurance coverage

## Abstract

**Background and objectives:**

Neuropsychiatric disorders are of high concern and burden of disease in the United Arab Emirates (UAE). The aim of this study is to describe patient cost-sharing patterns, insurance coverage of ambulatory neuropsychiatric disorders, and utilization of neuropsychiatric services in Abu Dhabi.

**Methods:**

The study utilized the data published by Health Authority-Abu Dhabi (HAAD) and the American Center for Psychiatry and Neurology (ACPN) records in Abu Dhabi. The data were collected from the ACPN to describe patterns of insurance coverage and patient cost-sharing. The data included information on patient visits to the ACPN from January 1, 2010 till May 16, 2013. The data also included insurance coverage, total cost of treatment for each patient and the amount of coinsurances and deductibles paid by each patient. Additionally, the study utilized data published by HAAD on health services utilization, and health insurance plans in 2014. The percentage of total costs paid by patients and insurance were calculated by insurance groups and health service. Insurance plans with different patient cost-sharing arrangements for mental health treatment benefits were divided into three groups. ANOVA and MANOVA analyses were performed to test for differences among three categories of neuropsychiatric services (neurology, psychiatry and psychotherapy) in terms of the total costs and patient cost-sharing. The data were analysed using STATA version 12.

**Results:**

About 36 % of the total costs on ambulatory neuropsychiatric services was paid directly by patients; 1 % of total costs was covered by patients as co-insurances and deductibles, and 63 % of total costs was covered by insurance providers. The average cost per visit was about 485 AED ($132), including 304 AED ($83) paid by insurance and 181 AED ($49) paid by patient. About 44 % of total costs was related to psychiatry services, 28 % of total costs was related to neurology services, and 28 % of total costs was related to psychotherapy services. Using ANOVA analyses, statistical differences were found among three categories of neuropsychiatric services in terms of the total costs and patient cost-sharing. These findings provide hint on some degree of association between patient cost-sharing and neuropsychiatry services utilization.

**Conclusions:**

The determination of parities in the coverage and finance between neuropsychiatric and physical health services will help policymakers make informed decisions on regulations of health insurance plans. Given the level of unmet need for neuropsychiatric services in Abu Dhabi, there is a need to fully include neuropsychiatric services in all basic and enhanced insurance plans. The study provided a description of patient cost-sharing and coverage of neuropsychiatric services in order for policymakers to recognize the disparities of the coverage and the degree of economic burden on households.

## Background

The United Arab Emirates (UAE) consists of seven Emirates and covers an area of about 83,600 square kilometres. The population of the UAE is made up of several different demographic groups from different cultural and socioeconomic backgrounds. According to United Nations estimates, the population of the UAE reached 9.3 million in 2013 [20]. The Emirate of Abu Dhabi (Abu Dhabi) is the largest emirate with an area of 67,340 square kilometres equivalent to 80 % of the total area of the UAE. In 2013, population of Abu Dhabi was estimated 2.73 million including 30 % females and 70 % males. About 18 % of the population are nationals and 82 % are expatriates. The median age is 19.6 years for nationals and 31.7 years for expatriates [16].

In 2013, the healthcare spending reached 62 billion AED ($16.8 billion) in the UAE, including 18 billion AED ($4.9 billion) in Abu Dhabi, and 10 billion AED ($2.7 billion) in the Emirate of Dubai (Dubai). Abu Dhabi introduced universal health insurance system in 2007 which covers almost all national population. The local government of Abu Dhabi is the key payer contributing about 77 % of total health expenditures (THE) [13]. The introduction of Thiqa insurance plan for nationals, combined with an expansion of private health insurance coverage, led to a dramatic reduction in out-of-pocket payments to reach only 3 % of THE in 2011. Employers’ contribution, through premium payments, accounted for 20 % of THE [10]. In Dubai, where universal health coverage will be effective by January 2016, households paid the highest rate in the Gulf Cooperation Council (GCC) countries at about 22 % of THE in 2012 out of their own pockets [8] compared to 16 % in the UAE [24].

Mental disorders constitute about 3 % of the global health budget and contribute to 13 % of the global burden of disease [23]. Neuropsychiatric disorders include neurological disorders as well as mental disorders. The estimated cost of neuropsychiatric disorders in the GCC countries in 2013 was about $9.43 billion; $1.68 billion as direct cost and $7.75 billion as indirect cost [4]. The direct cost translates to about 4.5 % of THE in the GCC countries. Abu Dhabi spent, in 2013, about 67 % of THE on ambulatory services, and 33 % on inpatient services. About 440 AED million (US$120 million) were spent on neuropsychiatric services which constitute about 4.4 % of THE including 52 % on ambulatory services and 48 % on inpatient services [16].

In the UAE, neuropsychiatric disorders are estimated to constitute 19.9 % of global burden of disease [21]. About 4–5 % of the UAE residents suffer from some form of mental or psychological disorder, including depression, anxiety, bipolar disorder, and schizophrenia [7]. Hamdan indicated that the prevalence of psychiatric morbidity in the Arab World was similar to that found in other parts of the world [12]. Al-Maskari et al. conducted a cross-sectional survey of depression and suicidal ideation among 239 migrant male workers. Twenty five percent (25 %) of the respondents suffered from depression, 6.3 % reported suicidal thoughts, and 2.5 % had attempted suicide. Depression and suicide ideation were associated with physical illness, working in construction industry, earning under $275 per month and having a long work shift—over 8 h per day [2].

Neuropsychiatric services are expected to grow in demand in Abu Dhabi [15], yet human resources are very limited. In 2011, there were only two psychiatrists per 100,000 population in Abu Dhabi compared to 16 psychiatrists per 100,000 population on average across the Organization for Economic Cooperation and Development (OECD) countries. In this emirate, there was only one nurse per 100,000 population compared to 50 mental health nurses per 100,000 population on average across OECD countries [15, 18]. It is estimated that in Abu Dhabi nearly 100,000 inhabitants suffer from mental disorders, but only 25,000 of those afflicted receive adequate treatment [26].

In Abu Dhabi, health insurance can be purchased directly from insurance companies or indirectly through insurance brokers. About 40 insurance providers, 53 insurance brokers, and 13 third party administrators offered three main types of health insurance plans: basic, Thiqa (trust) and enhanced. The basic plan covers expatriates with a total monthly salary package of under or equal to AED 4000 ($1090) with housing allowance, or 5000 AED ($1363) where housing is not provided by the employer [14]. Expatriates must enroll in the basic plan as mandatory minimum coverage offered at the government subsidized price of 600 AED ($163) per year, a premium that is determined by an executive decision from HAAD, the governing body of health care in Abu Dhabi. Basic plan is administered by National Health Insurance Company (Daman) and the other 17 insurance companies. Thiqa plan provides the UAE nationals working and residing in the Emirate of Abu Dhabi with comprehensive free coverage at all public and private facilities. Thiqa is administered by Daman and is regulated by HAAD. Expatriates have a choice of obtaining insurance coverage through purchasing an enhanced plan. To provide enhanced plan, insurance providers are required to make available a minimum of two significant enhancements in addition to what is offered in the basic plan, such as increasing the upper limit coverage, geographical area, and increasing inpatient and outpatient services. Most of the private plans do not cover neuropsychiatric disorders, unless these disorders are added at extra negotiated costs [17].

## Aims of the study

Although a multitude of factors affects access to neuropsychiatric services, lack of insurance coverage for mental health services and cost-sharing are two factors that merit further investigation. The aim of this *exploratory* study is to describe patient cost-sharing patterns and to provide insights into insurance coverage of mental health services and utilization of various types of ambulatory neuropsychiatric services in Abu Dhabi.

## Methods

The study utilized data published by HAAD and the American Center for Psychiatry and Neurology (ACPN) records in Abu Dhabi. The ACPN was founded in 2008 to meet the standards of high quality psychiatric and neurological services within the UAE. It is a premier medical facility focusing on providing primary and specialized neuropsychiatric services. The ACPN was used as a convenient sample of private neuropsychiatric health facilities. It is the second largest provider of ambulatory neuropsychiatric services in the Emirate of Abu Dhabi after Sheikh Khalifa Medical City (SKMC) Psychiatry unit; it has provided services to over 30,000 patients since its establishment [3]. During the period of study, the ACPN was the only center to provide comprehensive neuropsychiatric services in Abu Dhabi. Other providers provide limited psychiatric services.

The study analyzed the 2014 data by HAAD on health services utilization and three types of insurance health plans: Thiqa, basic, and enhanced. The data covering the time period, from January 1, 2010 till May 16, 2013, were also collected from the ACPN to describe patterns of insurance coverage of neuropsychiatric disorders, patient cost-sharing and utilization of mental health services. The administrative data selected for the purpose of this study included the number of patient visits to the ACPN, insurance coverage, the total cost of treatment for each patient and the amount of co-insurance paid by each patient. A descriptive analysis of the patient population was performed. Percentages of total costs paid by patients and insurance plans were calculated. ANOVA analyses were performed to test for statistical differences among the groups of the insurance plans with various cost-sharing arrangements. Average number of visits per patient and total treatment costs were used as measures of utilization of neuropsychiatric services.

To facilitate data analysis, insurance plans enrolees were classified intro three groups. The first group includes Thiqa plan which fully covers nationals for neuropsychiatric disorders. The second group includes enhanced plans which fully or partially covers expatriates for neuropsychiatric disorders. The last group, referred to as ‘basic plan’ includes basic plans that cover expatriates for neurology services, but do not provide any coverage for psychiatric disorders Neuropsychiatric services were also classified into three categories: neurology services, psychiatry services, and psychotherapy services. ANOVA and MANOVA analyses were employed to test for statistical differences in total costs and average cost per visit of three categories of neuropsychiatric services. The data were analysed using STATA version 12.

## Results

The basic plan partially covers about 1.34 million expatriate members in the Emirate of Abu Dhabi (41 % of insured population) for hospitalization, primary care, medical tests, dental care (excluding orthodontics and dentures), and prescription drugs. However, the plan does not cover psychiatric disorders, unless the condition is a transient or an acute reaction to stress. In 2014, there were approximately 300 enhanced plans which cover about 1.49 million members (44 % of insured population), offering full or partial coverage for physical health services. Only 2754 enhanced plans (18 % of all enhanced plans) offer full or partial coverage for neuropsychiatric services. Thiqa plan covers about 0.48 million national members (15 % of insured population), and provides full coverage for all physical health services and neuropsychiatric services as shown in Table 1.

**Table 1:** Coverage of medical and neuropsychiatric services by health plans

Plan type	Coverage of physical health services		Coverage of neuropsychiatric services	
	Nationals	Expatriates	Nationals	Expatriates
Basic	No	Partial	No	No except neurology services
Enhanced	No	Full or partial	No	Full or partial
Thiqa	Full coverage	No	Full coverage	No

Out of 2754 enhanced plans, only 896 plans offer full coverage of neuropsychiatric services without any user fee, and 1858 enhanced plans require considerable cost-sharing with limits on coverage outside the network and medicines. Out of 1858 enhanced plans, about 1369 plans require the deductible of 50 AED ($14) for general practitioner and specialist. About 415 enhanced plans require deductible ranging from 10 to 40 AED ($3–$11) for general practitioner and specialist; about 74 enhanced plans require co-insurance ranging from 5 to 50 % up to 50 AED ($14) per episode. Abu Dhabi health services company (SEHA) which is the main health provider in Abu Dhabi is able to apply to HAAD for emergency mental health services which are not covered by insurance to be covered under the Government Sponsored Health Services Policy [15].

Out of 896 enhanced plans which offer full coverage of neuropsychiatric services, 43 % (385 plans) cover 100 % of cost outside the provider network, 49 % (439 plans) cover 80 % or more of cost outside of network. Only 7 % (63 plans) cover less than 80 % of cost outside the network. 229 enhanced plans charge co-insurance ranging from 5 to 30 %.

From January 1, 2010 till May 16, 2013, 12,211 patients visited the ACPN in Abu Dhabi with about 57,824 ambulatory visits, an annual visit rate of about 1.6 visits per patient. About 66 % of patients were nationals, and 34 % were expatiates. 51 % of patients were females and 49 % were males. Approximately 3 % of visits made by children less than 5 years old; 4 % of visits made by elderly older than 65 years old. About 18 % of visits made by patients in their 20s; about 28 % of visits made by patients in their 30s; about 17 % of visits made by patients in their 40s. Depression was the most common illness among treated cases (8.6 % of patients), followed by anxiety state (4.6 %), attention deficit disorder with hyperactivity (3.1 %), and generalized anxiety disorder (2.8 %).

Total visits, total cost of neuropsychiatric services, and patient cost-sharing by insurance group are shown in Table 2.

**Table 2:** Total visits, total cost of neuropsychiatric services, and patient cost sharing by insurance group

Insurance group	No. of visits	Average no. of visits per patient	Total cost (AED)	Cost paid by patients AED (%)	Cost paid by insurance AED (%)
Thiqa	23,543	4.9	12,585,705	0	12,585,705 (100 %)
Enhanced	33,489	4.7	15,211,763	10,372,366 (68 %)	4839,397 (32 %)
Basic	792	4.3	258,728	78,375 (30 %)	180,353 (70 %)
Total	57,824	4.7	28,056,196	10,450,741 (37.2 %)	17,605,455 (62.8 %)

As shown in Table 2, 1.4 % of visits to the ACPN were made by basic plan holders who were not covered by insurance plans for psychiatric services. About 98.6 % of all visits were covered by insurance, as follows: 23,543 visits were covered by Thiqa plans and paid fully by insurance, 33,489 visits were covered by enhanced plans and either paid fully by insurance or paid jointly by patients and the insurance companies.

During the period of study, insurance companies covered 62.8 % of total costs of neuropsychiatric services and patients covered 37.2 % of the total costs on neuropsychiatric services by out-of-pocket payments. About 36.1 % of total costs were paid by patients as full cost payments and 1.1 % of total costs were paid as user fees (co-insurances and deductibles). Eleven insurance companies namely, ADCO Abu Dhabi, Abu Dhabi Gaz, ADNOC Distribution, Amity, Etihad Airways Medical Center, SHEPELL-FGI, ZADCO Abu Dhabi, international SOS, and Daman (Thiqa plan) insurance companies cover full cost of neuropsychiatric services. Other insurance companies covered the range of 86–98 % of the cost, so patient cost-sharing ranged from 2 to 14 % of total costs paid.

Nationals covered by Thiqa plan visited the ACPN more often than the expatriates covered by enhanced and basic insurance plans as measured by average number of visits.

Furthermore, the costs of neuropsychiatric services were broken down by the categories of services: neurology, psychiatry, and psychotherapy. The data summary is presented in Table 3.

**Table 3:** Cost and share (%) of three categories of neuropsychiatric services, by Group

Insurance group	Cost of neurology services AED (%)	Cost of psychiatry services AED (%)	Cost of psychotherapy services AED (%)
Thiqa	2926,319 (37 %)	7649,053 (62 %)	2010,333 (25.7 %)
Enhanced	4800,824 (61 %)	4650,829 (37.7 %)	5760,110 (73.8 %)
Basic	181,827 (2 %)	37,300 (0.3 %)	39,601 (0.5 %)
Total	7908,970 (100 %)	12,337,182 (100 %)	7810,044 (100 %)

Table 3 shows that about 62 % of the costs of psychiatry services was covered by Thiqa. Thiqa plan and enhanced plans covered 37 and 61 % of the costs of neurology services, respectively while basic plans covered only 2 % of neurology services. Those enrolled in basic plans incurred 39,601 (0.5 %) of the total costs of psychotherapy services.

Among the patients covered by Thiqa plan, 61 % of the total cost was related to psychiatric services. Among the patients covered by enhanced plans that fully or partially cover neuropsychiatric disorders, 38 % of the total cost was related to psychotherapy services. Among the patients enrolled in the basic plans that do not cover psychiatric disorders, 70 % of the total cost was related to neurology services. In Abu Dhabi, neurology services are covered by most insurance companies while psychiatric and psychological services have limited coverage. In total, for all three Groups, 44 % of the cost was related to psychiatry, 28 % related to neurology, and 28 % related to psychotherapy. However, as mentioned above, the largest portion of the costs of psychiatry disorders (62 %) were paid through Thiqa plan for nationals, as shown in Table 3. Most of the expatriate patients contributed either fully or partially to the cost of neuropsychiatric services they received. Basic plans enrolees paid 37,300 AED out of pocket for psychiatry services with no contribution from insurance. However, insurance contributed 169,807 AED for neurology services of basic plan enrolees as shown in Table 4.

**Table 4:** Patient cost-sharing for three categories of neuropsychiatric services for basic plans group

Categories of neuropsychiatric services	Payment by patient (AED)	Payment by insurance (AED)
Psychiatry	37,300	0
Psychotherapy	29,055	10,546
Neurology	12,020	169,807
Total	78,375	180,353

It was estimated that the average cost per visit was about 485 AED ($132). The average cost per visit paid by patient was about 181 AED ($49). The average cost per visit paid by insurance was about 304 AED ($83). Income per capita in Abu Dhabi was about $43,048 in 2013. The cost of a visit to neurology department ranked the highest with an average cost of 533 AED ($145) per visit as shown in Table 5.

**Table 5:** The average cost per outpatient visit by categories of neuropsychiatric services

Categories of neuropsychiatric services	Total cost AED (%)	Average cost per visit paid by patient AED (US $) (%)	Average cost per visit paid by insurance AED (US $) (%)	Average cost per visit AED (US $) (%)
Psychiatry	12,337,182 (44 %)	151 ($41) (34 %)	300 ($82) (66 %)	451 ($123) (100 %)
Neurology	7908,970 (28 %)	75 ($20) (14 %)	458 ($132) (86 %)	533 ($145) (100 %)
Psychotherapy	7810,044 (28 %)	335 ($91) (67 %)	165 ($45) (33 %)	500 ($136) (100 %)
Total	28,056,196 (100 %)	181 ($49) (37 %)	304 ($83) (63 %)	485 ($132) (100 %)

Results of ANOVA analysis showed that the mean of average costs paid by patients, representing patient cost-sharing, and mean of total costs are statistically significantly different among three categories of neuropsychiatric services, (F = 1155, p < 0.0001) and (F = 115.2, p < 0.001), respectively. Results of MANOVA analysis showed that mean differences among psychiatry, neurology and psychotherapy on a combination of patient costs and insurance costs were not likely to occur by chance, and the differences are significant. The multivariate test of differences among the three types of services using the Wilks Lambda criteria was statistically significant [F (4, 115,640) = 1654.7; p < 0.00]. Follow-up multivariate comparisons showed that the average cost of neurology service was significantly different from the average cost of psychiatry and average cost of psychotherapy [F (2, 57,820) = 1592; p < 0.00]. Further, it was determined that the average cost of psychiatry service and the average cost of psychotherapy were also significantly different [F (2, 57,820) = 3186.23; p < 0.00]. The multivariate test was rerun using the combination of average cost of neurology service and average cost of psychotherapy service, which was statistically significant [F (2, 57,820) = 3186; p < 0.00].

## Discussion of the results

A few important issues came out of the results: (1) disparity in insurance coverage and access to services for the UAE nationals and expatriates; (2) disparity in insurance coverage between neuropsychiatric and physical health services, (3) potential association between patient cost-sharing and utilization of neuropsychiatric services.

### Description of patient population

The study indicated that about 51 % of patients were females compared to 28 % of the total population, and 49 % were males compared to 72 % of the total population. Women, in particular, are more likely to suffer from various neuropsychiatric disorders including depression, anxiety disorders, somatization, and eating disorder [12]. About 18 % of visits made by patients in their 20s, about 28 % of visits made by patients in their 30s, and about 17 % of visits made by patients in their 40s. Children and the elderly appear to have lower rates of utilization compared with general population. About 3 % of visits made by children less than 5 years old and 4 % of visits made by elderly older than 65 years old.

The study found that depression was the most common diagnosis among treated cases (8.6 % of patients), followed by anxiety state (4.6 %), attention deficit disorder with hyperactivity (3.1 %), and generalized anxiety disorder (2.8 %). Such estimates have drawn attention to the importance of neuropsychiatric disorders for public health in Abu Dhabi. This is in line with global and regional view highlighting that depression alone accounts for 4.3 % of the global burden of disease, particularly for women [25]. Treatment of depression and other mental disorders should be regarded as an investment to reduce health care costs and disease burden, and to improve quality of life [5].

### Disparity in insurance coverage and access to services for UAE nationals and expatriates

The demand for neuropsychiatric services is believed to be responsive to the terms of insurance coverage. The findings of this study indicated that about 66 % of neuropsychiatry patients were nationals while they constitute 18 % of total population, and 34 % of patients were expatiates while they constitute 82 % of the total population. The study found that nationals, fully covered by Thiqa plan for neuropsychiatric disorders, accounted for 45 % of the total costs on ambulatory neuropsychiatric services utilized during the period of the study, while expatriates enrolled in the basic and enhanced plans, which cover 85 % of the population, accounted for 55 % of the total costs (Table 2). As expected, nationals utilized more neuropsychiatric services than expatriates relative to their share of the total population. This conclusion is also confirmed by the fact that the annual visit rate among the treated patients was about 2.3 visits per patient for nationals compared to about 1.1 for expatriates. Similar conclusion was reported by Hamidi et al. [14] for physical health services in Abu Dhabi [14].

Cost-sharing might decrease demand for neuropsychiatric services. In our study population, average number of visits per patients was largest in Thiqa group and smallest in Basic group. The highest number of visits were made by enhanced group. Those enrolled in the basic plans made only 792 out of 57,824 visits corresponding to 1.4 % of all visits. Expatriates with no insurance coverage for mental health might have viewed lack of insurance coverage as a barrier to access mental health services. Even though some basic plan holders received a discount for psychiatry services at the ACPN upon presentation of salary certificates, very limited number of basic plans enrolees accessed such services—mainly those who first presented with neurological disorders covered by all insurance plans and were later referred within the same facility to a psychiatrist. Expatriates cannot access publicly-funded institutions providing neuropsychiatric services; their choices are limited to the private ones such as the ACPN. Access to mental health care is available for free to Emiratis covered by Thiqa at public and private locations.

### Disparity in insurance coverage between neuropsychiatric and physical health services

Abu Dhabi has universal health coverage, however insurance coverage does not guarantee access to services, and disparity exists between neuropsychiatric and physical health services in terms of insurance coverage. Insurance coverage of neuropsychiatric services has been less extensive than care for physical health conditions in Abu Dhabi. About 82 % of enhanced plans for expatriates cover fully and partially physical health services. However, only 12 % of enhanced plans partially cover and 6 % fully cover neuropsychiatric services.

Disparity in coverage between neuropsychiatric and physical health services is not unique to the UAE. Some degree of disparity, to a lesser extent, have been reported in Europe and North America. For example, the USA has different prior authorization requirements, higher cost-sharing structures, and limits on inpatient and outpatient visits for mental health care [11]. Acceptance rates for all types of insurance, such as private non-capitated, Medicare or Medicaid, were significantly lower for psychiatrists than for physicians in other specialties [6]. However, the Affordable Care Act (ACA) introduced in 2010, aimed to improve mental health parity by establishing mental care as an essential health benefit, applying federal parity rules to ensure that coverage is comparable. In European countries disparity in coverage of mental health and physical health varies by country. In Sweden, mental health is an integrated part of the health system and is subject to the same legislation and user fees as other health services [19]. Psychotherapy can be accessed in hospitals with public funds contributing to at least 80 % of the cost in 81 % of European countries. Patient can receive psychotherapy services in community settings in 64 % of European countries [22].

Interestingly, neuropsychiatric disorders were estimated to contribute to about 20 % of the global burden of disease in Abu Dhabi, yet they constitute only 4.4 % of health expenditures, compared to 16 % in France [16, 19]. Mental care is an integral part of the National Health Service (NHS) in the UK, including a full range of services, with prescription drugs covered under the same terms as other NHS drugs. Abu Dhabi would benefit from legislation similar to Mental Health Act of the UK, which is a code of practice to protect those with mental disorders. Any meaningful approach to health must entail parity in financing physical and mental health services.

### Potential association between patient cost-sharing and utilization of neuropsychiatric services

Using administrative data from the Guangzhou Psychiatric Hospital, Zhou et al. examined relationship between variation in depth of insurance coverage and psychiatric hospital utilization. Multiple logistic regression analyses were used, and important confounders such as demographic characteristic and medical diagnoses were controlled for. They found that insurance plans with lower co-payments were significant predictors of multiple admissions to psychiatric hospital and longer length of stay [27].

Our study provides some insights into potential association between cost-sharing and mental health services utilization. The nationals fully covered by Thiqa insurance for neuropsychiatry services had higher average number of visits compared to other two groups comprised of expatriates. Moreover, utilization patterns of three categories of neuropsychiatric services, measured by costs of services provided, varied among three insurance groups with various degrees of patient cost-sharing.

Patients enrolled in Thiqa utilized mainly psychiatry services which were fully covered by insurance. (Table 3). Expatriates enrolled in enhanced insurance plans, with either full or partial insurance coverage for mental health services, utilized large portion of psychotherapy services. Lastly, expatriates enrolled in basic plans, who had no insurance coverage for psychiatry services, utilized more neurology services as measured by costs of services for this group. This can be explained by possible cost-sharing barriers in accessing psychiatry and psychotherapy services by basic group enrollees or different mental health patterns of this group compared to the other group of expatriates with higher earnings.

It is important to note that basic plans enrollees had relatively small total number of visits to the ACPN (792 out of 52,824) during the study period. This may indicate probable underutilization of services due to cost-sharing barriers. There are no charitable or community-based neuropsychiatric services available to expatriates; they can seek treatment only in private facilities. During the period of study, the ACPN was the only center to provide comprehensive neuropsychiatric services in Abu Dhabi. The center has no waiting time; patients can receive same day services from the specialists. The ACPN employs the majority of psychiatrists in Abu Dhabi.

To the best of our knowledge, this is the first study of its kind in the UAE. We employed a descriptive approach to find some hints on possible associations between some patient-related and health economic factors. The interpretation of these results, however, must take into consideration three limitations. Firstly, this study was based on the data collected from the ACPN center in Abu Dhabi and the data collected from HAAD insurance plans database. The ACPN patient population was chosen as a convenient sample and may not fully represent the Emirate of Abu Dhabi. However, it is important to note that the ACPN is the second largest provider of neuropsychiatric services in Abu Dhabi in terms of the number of patients served. During the period of the study, the ACPN and Sheikh Khalifa Medical City were the only mental health facilities which provided full scope ambulatory mental health services to the patient population of all ages and sexes in the Emirate of Abu Dhabi. Secondly, limitations in the data hindered our ability to control for important potential confounders, including socio-demographic characteristics and neuropsychiatric diagnosis. Thirdly, a large proportion of the ACPN patients were nationals fully covered by Thiqa plan. Therefore, Thiqa plan contributed to a major proportion of the total costs. Nonetheless, our study provides facts and perspective to important barriers affecting the use of mental health services specific to our region and population.

## Conclusions

Only about 18 % of health insurance plans cover neuropsychiatric services in Abu Dhabi. About 33 % of those 18 % offer full insurance coverage and without any user fee. The remaining 67 % of the plans require deductible or co-insurance. Given the level of unmet need for neuropsychiatric services in Abu Dhabi, there is a need to fully include neuropsychiatric services in all basic and enhanced insurance plans.

There is a possibility that the number of people requiring neuropsychiatric services in the Emirate of Abu Dhabi is under reported. Some patients do not seek care and choose to self-pay for treatment because of the social stigma associated with neuropsychiatric illness. Moreover, studies on mental health status in the Arab world found large proportions of respondents who were turning to traditional healers and prayer during times of psychological distress [1, 9]. Therefore, development and implementation of screening for neuropsychiatric health are crucial steps to uncover the potential of the problem. Furthermore, providing insurance coverage to ambulatory neuropsychiatric services continues to be an important enabling factor in obtaining care, but is an incomplete solution to assuring that needed health care services are obtained. High patient cost-sharing presents a major obstacle to seeking care. Although enhanced health insurance plans have attempted to eliminate some financial barriers to neuropsychiatric health services, these plans may not have funded services as generously as neuropsychiatric health needs demand. In this aspect, the private sector has a role to play, and the commitment of policymakers is crucial for the prioritization and integration of private sector into neuropsychiatric health delivery system. Any meaningful approach to health must entail parity in financing physical and mental health services.

This exploratory study was designed to describe and analyze the patient cost-sharing and insurance coverage of ambulatory neuropsychiatric services in Abu Dhabi. The findings point to potential opportunities for reform in health policy regarding the comprehensiveness of neuropsychiatric coverage and the scope of patient cost-sharing. In order to meet the growing need of mental health in Abu Dhabi, it is important to provide a clear description of patient cost-sharing and coverage of neuropsychiatric services in order for policymakers to recognize the disparities of the coverage and the degree of economic burden on households. This knowledge will help with making informed decisions on regulations of health insurance plans.

The goal of this study was to translate the data collected by the HAAD and ACPN into policy relevant information; the study is indeed the very first step towards achieving more equitable coverage, and providing well-needed locally relevant research. Indeed, this study suggests a promising avenue for neuropsychiatric health services research. This article is an invitation to other researchers in the field to identify barriers to access neuropsychiatric health services, to estimate the effect of cost-sharing on the utilization of inpatient neuropsychiatric services, to analyze the impact of patient cost-sharing on outcomes and use of post-discharge outpatient care, and to examine the effect of cost-sharing on the choice of different providers. These procedures will help untangle the complex web of factors that inhibit appropriate and timely access to health services.
